# Ultrastructure of Myzocytosis and Cyst Formation, and the Role of Actin in Tubular Tether Formation in *Colpodella* sp. (ATCC 50594)

**DOI:** 10.3390/pathogens11040455

**Published:** 2022-04-11

**Authors:** Tobili Y. Sam-Yellowe, Hisashi Fujioka, John W. Peterson

**Affiliations:** 1 Department of Biological, Geological and Environmental Sciences, Cleveland State University, Cleveland, OH 44115, USA; 2 Cryo-EM Core, Case Western Reserve University, Cleveland, OH 44106, USA; hxf3@case.edu; 3 Cleveland Clinic Lerner Research Institute, Cleveland, OH 44195, USA; petersj@ccf.org

**Keywords:** apicomplexa, *Colpodella* sp. (ATCC 50594), myzocytosis, *Colpodella* ultrastructure, *Colpodella* cysts, trichrome stain, rostrum, pseudoconoid, actin stain, cytochalasin D

## Abstract

Free-living relatives of the Apicomplexa such as *Colpodella* species, *Alphamonas* species, and *Voromonas pontica* are predators that prey on ciliate, bodonid, and algal prey using the process of myzocytosis. During myzocytosis, the pseudoconoid is used to attach to the prey leading to aspiration of cytoplasmic contents of the prey into a posterior food vacuole formed in the predator, aided by secretions from the apical complex organelles. The conoid and associated proteins are conserved among the apicomplexa. However, the organization and function of the pseudoconoid during myzocytosis are not well understood. In this study, we investigated the morphology and ultrastructure of *Colpodella* sp. (ATCC 50594) during the stages of myzocytosis and cyst formation in the life cycle using light microscopy and transmission electron microscopy (TEM) in order to identify the organization of the tubular tether involved in nutrient aspiration by *Colpodella* sp. Tubular tethers of varying lengths were identified by light microscopy. We report that initial contact by *Colpodella* sp. trophozoites with *Parabodo caudatus* prey is by an area posterior to the apical tip of the rostrum that engulfs the membrane of the prey pulling it into the cytoplasm of the predator. The tubular tether that forms contains membranes of both predator and prey and is facilitated by microtubule organization and the cytoskeleton at the point of contact. Cytochalasin D treatment of diprotist cultures resulted in morphological distortions of trophozoites and the tubular tether suggesting a role of actin in the formation of the tubular tether. This mechanism of predation may provide insight into the mode of invasion observed in pathogenic apicomplexan zoites during host cell entry.

## 1. Introduction

Apicomplexans comprise important pathogens causing human and animal infections such as malaria, toxoplasmosis, and babesiosis. The mechanism of invasion among pathogenic apicomplexans involves direct contact of the invasive stages (zoites) with the host cell using the apical end [1,2,3]. This is followed by the secretion of proteins from the apical complex organelles such as the rhoptries and micronemes to facilitate invasion and early development of the intracellular parasite within the host cell [1,4]. Free-living alveolate relatives of the apicomplexans such as *Colpodella* species, *Chromera velia*, *Vitrella brassicaformis*, and *Voromonas pontica* have also been described and share the features found in the apical complex [4,5,6,7,8]. While the role of the apical complex remains unclear among the photosynthetic free-living apicomplexans, the mode of budding described in *C. velia* and *V. brassicaformis* and the process of microgametogenesis are considered ancestral forms of schizogony and endodyogeny and microgamete development in the Apicomplexa [4,9]. Among the dinoflagellates, *Perkinsus* and *Psammosa* species possess an apical complex with a pseudoconoid, rhoptries, and micronemes [10,11,12]. Apicomplexan free-living relatives carry out myzocytosis, which is a form of endocytosis [13]. The apical complex is used for myzocytosis among these diverse lineages, and among the colpodellids, a tubular tether is formed between predator and prey [4,5,6,7,8]. In *Chromera velia*, in addition to being photosynthetic, the apical complex is thought to be instrumental in establishing endosymbiosis [12]. Feeding through myzocytosis involves the use of the mucron at the apical tip of the trophozoite stages in aconoidasida, such as in the gregarines [4,14]. The tip of the pseudoconoid contained within a rostrum in the free-living colpodellids is described as the point of contact with prey to initiate myzocytosis [4,14]. Archigregarines employ myzocytosis for nutrient uptake and are thought to bridge the progression of myzocytosis in the predatory colpodellids to epicellular parasitism, which is also seen in gregarines [14]. The feeding and invasive mechanisms in Apicomplexa are thought to have progressed from epicellular parasitism present in gregarines and in *Cryptosporidium* species to intracellular parasitism found in pathogenic Apicomplexa such as in *Plasmodium* species, *Toxoplasma gondii*, and other coccidia [4,14]. The origins of extracellular and intracellular parasitism can be found in myzocytosis described in diverse lineages of Alveolates [14]. Following myzocytosis in cyst-forming *Colpodella* species, the trophozoite with the food vacuole and nucleus can be recognized. The *Colpodella* sp. (ATCC 50594) trophozoite, containing aspirated contents from the prey and the cell nucleus, differentiates into a cyst stage that divides to produce asynchronous symmetric or asymmetric trophozoite progeny [5,6,7,15]. Resting and reproductive cyst-forming *Colpodella* species include *Colpodella* sp. (ATCC 50594), *C. vorax*, *C. tetrahymenae*, *C. pugnax*, and *C. turpis* [5,6,7,15]. *Colpodella pseudoedax* divides by longitudinal fission and only produces resting cysts [8]. *Colpodella* species have been implicated in two cases of human infection, characterized by neurological symptoms and babesiosis-like symptoms [16,17]. The mode of human infection and pathogenesis among the opportunistic *Colpodella* species has not been investigated. The life cycle stages of *Colpodella* sp. within the human host cells have not been described and the stages responsible for pathogenesis are unknown [16,17]. *Colpodella gonderi* was identified in urine samples in a case of urinary tract infection [18] although the protist did not appear to be the etiological agent of the infection. *Colpodella* species identified in animal hosts and in ticks have also been described, but characteristics of the identified organisms are unknown [19,20,21,22]. The characteristic apical complex organelles and cytoskeleton for which the phylum is named are found in both free-living and pathogenic members of the phylum. Rhoptries and micronemes have been identified, and a pseudoconoid housed within a rostrum was also identified in *Colpodella* species [5,6,7,8]. Previous ultrastructural studies identified *Colpodella vorax*, *Colpodella gonderi*, *Colpodella tetrahymenae*, and *Colpodella* sp. (ATCC 50594) trophozoites, cysts, and cells attached in myzocytosis [6,7,23,24,25,26], and identified the involvement of microtubules at the point of attachment [6,7,26]. In this study, we investigated the ultrastructure of the tubular tether and cyst stages of *Colpodella* sp. (ATCC 50594) and investigated the role of actin in the formation of the tubular tether. We used staining and light microscopy to demonstrate tubular tethers with varying lengths and to identify the effects of Cytochalasin D on the tubular tethers. We performed ultrastructural studies to further our understanding of the organization of the point of attachment and the formation of the tubular tether. We show that the tubular tether consists of the membranes and cytoskeleton of both predator and prey. Following attachment of the predator to prey, the prey’s membrane is not pierced but is engulfed into the cytoplasm of the predator before the prey’s membrane is destroyed and cytoplasmic contents are aspirated into the posterior food vacuole of the predator.

## 2. Results and Discussion

### 2.1. General Staining

**Figure 1:**
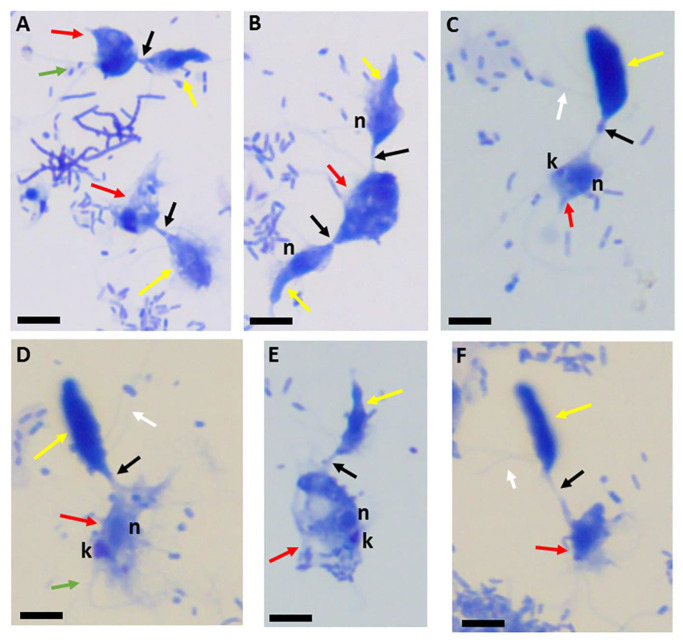
Giemsa-stained formalin-fixed *Colpodella* sp. (ATCC 50594) (yellow arrow) and *Parabodo caudatus* (red arrow) in myzocytosis showing different lengths of the tubular tether formed between both protists (black arrow). The flagella of *Colpodella* sp. (ATCC 50594) are indicated by the white arrows and flagella for *P. caudatus* by the green arrow. Attachment can occur with a single trophozoite of *Colpodella* sp. (ATCC 50594) attached to *P. caudatus* (**A**,**C**–**F**) or *P. caudatus* can be attacked by two *Colpodella* sp. (ATCC 50594) trophozoites at the same time as seen in panel (**B**). Scale bars, 10 µm.

In previous studies, we developed staining protocols to aid the identification of life cycle stages of *Colpodella* sp. (ATCC 50594) [15,24]. In particular, the cyst stages of *Colpodella* sp. (ATCC 50594) could be identified. In reports of human infections caused by *Colpodella* species, infective stages were not described [16,17] and the identification of additional life cycle stages in the urine of a patient shown to have *Colpodella gonderi* was not reported [18]. The staining protocols using Giemsa and Kinyoun’s carbol fuchsin identify the trophozoites and cysts of predator and prey and identify predator and prey in myzocytosis. The use of Sam-Yellowe’s trichrome staining further differentiates life cycle stages such as the cyst stages to identify immature and mature cysts. The use of staining protocols for light microscopy was not used in previous studies investigating the morphology and biology of *Colpodella* species [5,6,7,8,23]. In the present study, we investigated the ultrastructure of the tubular tether formed during myzocytosis and sought to determine the role of actin in the formation of the tether. Furthermore, we investigated the ultrastructure of the early cyst stages of *Colpodella* sp. (ATCC 50594). Formalin-fixed cells of *Colpodella* sp. (ATCC 50594) stained with Giemsa, Kinyoun’s carbol fuchsin, and Sam-Yellowe’s trichrome stains identified the tubular tether with varying lengths as shown previously [24]. Figure 1 shows Giemsa staining used to visualize trophozoites of *Colpodella* sp. (ATCC 50594) (yellow arrow) in myzocytosis with the prey *P. caudatus* (red arrow). Different lengths of the tubular tether are shown (black arrows). The flagella of the predator (white arrows) and prey (green arrows) were identified. Single *Copodella* sp. (ATCC 50594) attachments to prey (Figure 1A,C–F) and attacks by two *Copodella* sp. (ATCC 50594) trophozoites are shown (Figure 1B). Two major life cycle stages occur in *Colpodella* species, which are cysts and trophozoites. The trophozoites have two heterodynamic flagella.

**Figure 2:**
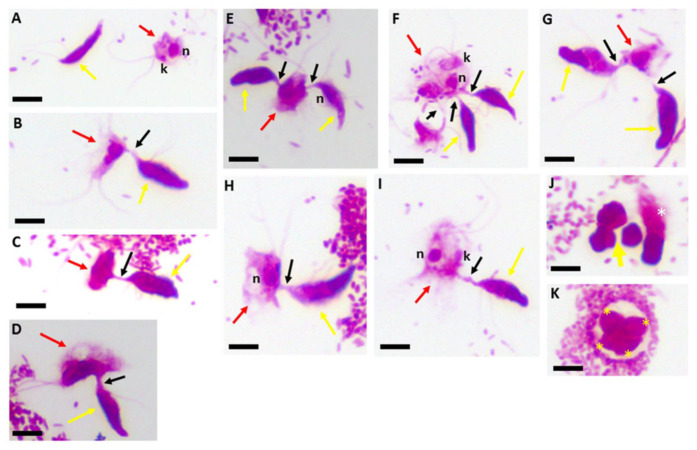
Kinyoun’s carbol fuchsin-stained formalin-fixed *Colpodella* sp. (ATCC 50594) (yellow arrow) and *Parabodo caudatus* (red arrow) shown individually (**A**), in myzocytosis showing different lengths of the tubular tether formed between the two protists and the flagella of both protists. Single attachments are shown in panels (**B**–**D**,**H**,**I**). *Parabodo caudatus* attacked by two trophozoites of *Colpodella* sp. (ATCC 50594) show that predators can attach to prey in close proximity or on opposite sides of the prey (**E**–**G**). Demilune cysts of *Colpodella* sp. (ATCC 50594) are shown in panel (**J**) (yellow arrow) and a pre-cyst showing the frayed anterior end of the trophozoite (white asterisk) is shown. A mature cyst of *Colpodella* sp. (ATCC 50594) containing four trophozoites (yellow asterisks) and a clear zone surrounding the cyst, free of bacteria is shown in panel (**K**). Scale bars, 10 µm.

The cysts are non-motile. Figure 2 shows Kinyoun’s staining of trophozoites of both predator and prey showing the flagella for both trophozoites of predator and prey (Figure 2A) and the different lengths of the tubular tether formed during myzocytosis including the flagella of both predator and prey.

Single attacks (Figure 2B,C,H,I) and predators attached in close proximity or on opposite sides of the prey are shown (Figure 2E–G). Demilune cysts of *Colpodella* sp. (ATCC 50594) are shown in Figure 2J (yellow arrow) and a precyst (white asterisk) is shown. A mature cyst of *Colpodella* sp. (ATCC 50594) with four trophozoites and a clear zone free of bacteria is shown (Figure 2K). The advantage of using Giemsa and Kinyoun’s carbol fuchsin is the identification of the nuclei and flagella of both protists and the kinetoplast of the prey [26]. The single blue and red colors of Giemsa and Kinyoun’s staining, respectively, do not allow for differentiation of developmental stages. Sam-Yellowe’s trichrome stain was used to visualize and differentiate trophozoite and cyst stages of *Colpodella* sp. (ATCC 50594). Trophozoites (yellow arrow) are shown in myzocytosis with *P. caudatus* (red arrow).

**Figure 3:**
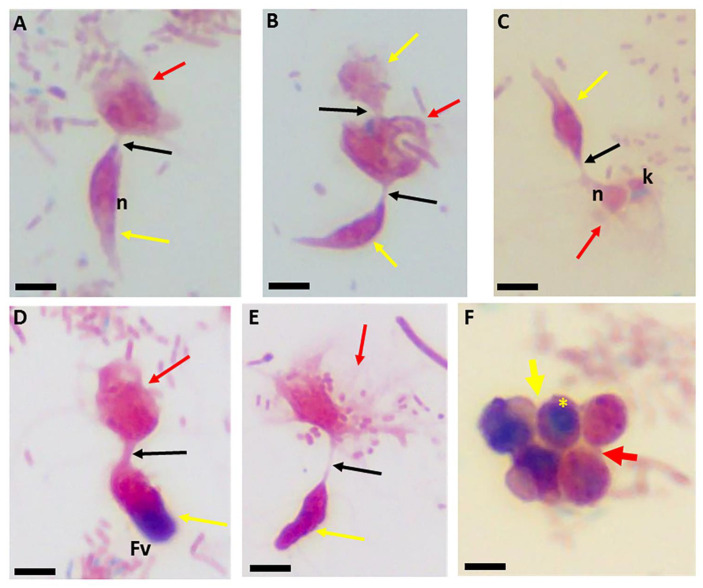
Sam-Yellowe’s trichrome-stained formalin-fixed *Colpodella* sp. (ATCC 50594) (yellow arrow) and *Parabodo caudatus* (red arrow) in myzocytosis showing different lengths of the tubular tether and differentiation of cyst stages. Single predator attacks are shown in panels (**A**,**C**–**E**). A single prey attacked by two predators is shown in panel (**B**). The nuclei (n) of both protists are shown in panels (**A**,**C**). The kinetoplast (k) of *Parabodo caudatus* is shown in panel (**C**). An enlarged darkly stained food vacuole in *Colpodella* sp. (ATCC 50594) is seen in panel (**D**). Demilune and single nucleus (yellow asterisk) cysts are shown in panel (**F**). *Parabodo caudatus* cysts (red arrow) can be distinguished from *Colpodella* sp. (ATCC 50594) cysts. The single nucleus cyst of *Colpodella* sp. (ATCC 50594) indicated by the yellow asterisk can be distinguished from the immature cysts of *Colpodella* sp. (ATCC 50594) showing partly light and dark stained sections in the cyst (yellow arrow). Scale bars, 10 µm.

**Figure 4:**
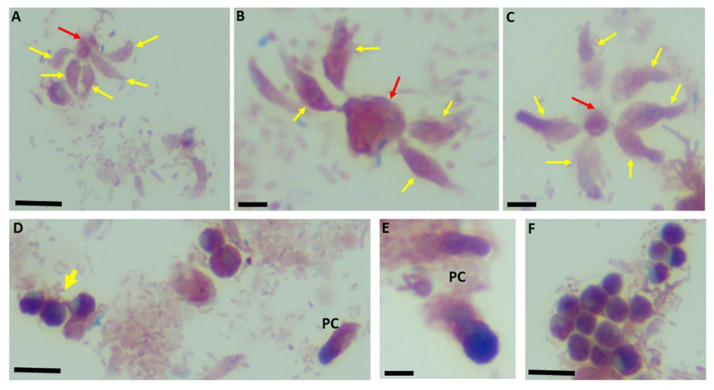
Sam-Yellowe’s trichrome staining of formalin-fixed *Colpodella* sp. (ATCC 50594) trophozoites (yellow arrows) attached to *P. caudatus* (red arrow). Multiple predators are shown attached to a single *P. caudatus* prey (**A**–**C**). Demilune cysts of *Colpodella* sp. (ATCC 50594) are shown in panels (**D**,**F**). Pre-cysts (PC) of *Colpodella* sp. (ATCC 50594) are shown in panels (**D**,**E**). Scale bars, 10 µm in panels (**B**,**C**,**E**). Scale bars, 15 µm in panels (**A**,**D**,**F**).

The use of staining for light microscopy is not routinely performed for the identification of the free-living apicomplexan relatives. In previous studies [24], and in the current study, we show that light microscopy of stained cells facilitates the interpretations of ultrastructural data. Different lengths of the tubular tether formed during myzocytosis (black arrow) are shown. The nucleus (n) of both protists is shown in Figure 3A,C. The kinetoplast (k) of *P. caudatus* is shown in Figure 3C. An enlarged posterior food vacuole of *Colpodella* sp. (ATCC 50594) trophozoite, darkly stained, is seen in Figure 3D. Demilune and single nucleus cysts (yellow asterisk) are shown in Figure 3F. Cysts of *P. caudatus* (red arrow) can be distinguished from *Colpodella* sp. (ATCC 50594) cysts. During myzocytosis, the predator aspirates the cytoplasmic contents out of its prey and into a posterior food vacuole. Multiple predators can attach to a single prey in the most active phase of the in vitro life cycle as shown with Sam-Yellowe’s trichrome staining in Figure 4A–C. This time period corresponds to the encystment of the prey in culture [24].

**Figure 5:**
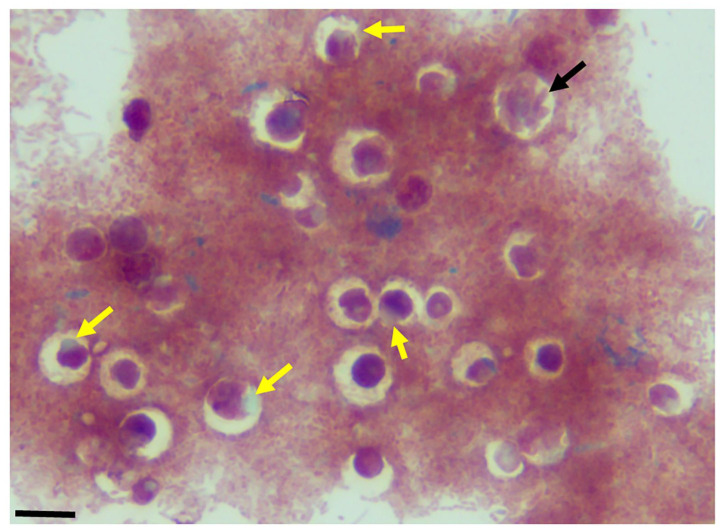
Sam-Yellowe’s trichrome-stained formalin-fixed cysts of *Colpodella* sp. (ATCC 50594) (yellow arrow) showing clear bacteria-free zones surrounding cysts. A mature cyst of *Colpodella* sp. (ATCC 50594) containing multiple juvenile trophozoites is shown (black arrow). Scale bars, 15 µm.

In previous studies, the most active phase of the life cycle of *Colpodella* sp. (ATCC 50594) in vitro was shown to be between 20 and 28 h [24]. With the early encystment of *P. caudatus* in the in vitro life cycle resulting in few trophozoites, multiple predators can be identified attached to single *P. caudatus* trophozoites in close proximity on the surface of the prey as shown in our previous studies [24] and in the current study. Demilune cysts of *Colpodella* sp. (ATCC 50594) are shown in Figure 4D,F. Precysts (PC) of *Colpodella* sp. (50594) are shown in Figure 4D,E. The precyst stage is formed following the feeding of the *Colpodella* sp. (ATCC 50594) trophozoite [24]. The anterior end of the trophozoite becomes frayed leaving behind the food vacuole and nucleus [24]. Bacteria-free zones are shown around cysts of *Colpodella* sp. (ATCC 50594) in Figure 5. These zones have only been observed around *Colpodella* sp. (ATCC 50594) cysts and not around *P. caudatus* cysts [15,24] The developing cysts in the presence of the bacteria present in the diprotist culture may be secreting antimicrobial products to protect the developing cyst. The data showing these bacteria-free zones is presently unclear and requires additional investigation. Similar bacteria-free zones around *Colpodella* species cysts have not been reported previously. A mature cyst with multiple juvenile trophozoites is shown (black arrow).

Further investigations will demonstrate if the clear zones are the result of antimicrobial activity or artifacts of fixation. The clear zones are identified only around *Colpodella* sp. (ATCC 50594) cysts, both immature and mature cysts, and not around *P. caudatus* cysts [15,24].

### 2.2. Ultrastructure of Tubular Tether and Cysts

**Figure 6:**
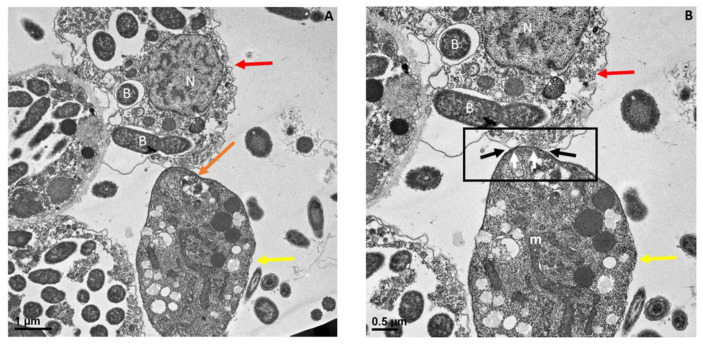
Transmission electron microscopy showing attachment site of *Colpodella* sp. (ATCC 50594) trophozoite (yellow arrow) to *P. caudatus* (red arrow). The attachment site is posterior to the apical tip of the rostrum indicated by the orange arrow (**A**). (**B**) shows the attachment site in an enlargement of panel (**A**) showing a two-point attachment (black arrows in boxed area). The white arrows identify the membrane of *Colpodella* sp. (ATCC 50594 at the attachment site. Bacteria (B) in the cytoplasm of *P. caudatus* are shown. Mitochondria (m) were also detected. Scale bars, (**A**) 1 µm and (**B**) 0.5 µm.

Myzocytosis between *Colpodella* species and their prey have been described using transmission electron microscopy [6,7,23,25,26]. However, the details of the attachment, food vacuole formation, and the differentiation of the food vacuole leading to cyst formation from early cysts stages to mature cysts are unknown. We reported the progression of these life cycle stages previously and described the life cycle of *Colpodella* sp. (ATCC 50594) in vitro [24]. In the current study, we wanted to know if the tubular tether observed by staining for light microscopy (Figure 1, Figure 2 and Figure 3), showing different lengths of the tether, was a product of the extension of the plasma membrane of *Colpodella* sp. (ATCC 50594) trophozoites in the apical end of the trophozoite for attachment to prey. The ultrastructure of the attachment site and tubular tether was investigated. Figure 6 shows that the attachment site on *Colpodella* sp. (ATCC 50594) (yellow arrow) is not at the apical tip (orange arrow) but in an area posterior to the apical tip of the rostrum (Figure 6A).

**Figure 7:**
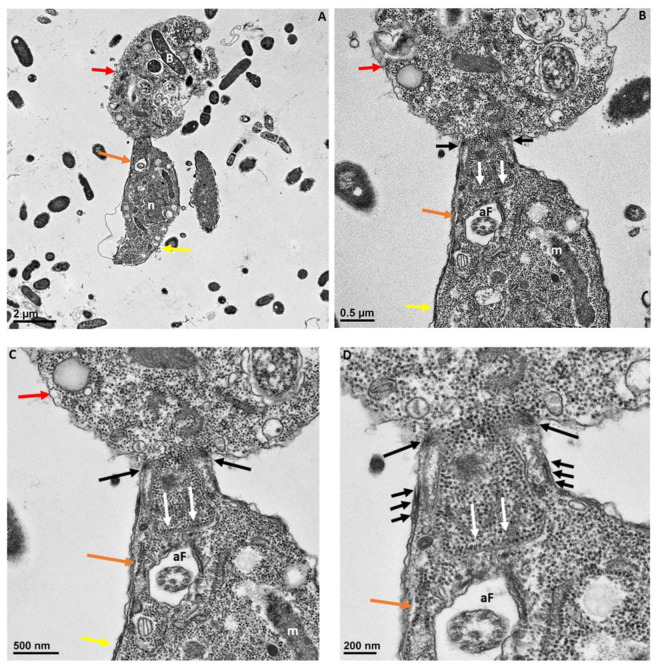
Transmission electron microscopy showing attachment of *Colpodella* sp. (ATCC 50594) trophozoites (yellow arrow) to *P. cuadatus* (red arrow). Panel (**A**) is enlarged in panels (**B**–**D**) to show the detail at the attachment site. The plasma membrane and cytoplasm of the prey are aspirated into the cytoplasm of the predator. White arrows show the prey’s plasma membrane. The attachment site is posterior to the apical tip (orange arrow) of the rostrum. Microtubules organized at the attachment zone (black arrows) are shown. Panels (**C**,**D**) show details of the attachment site and the tubular tether consisting of membranes of *Colpodella* sp. (ATCC 50594) and *P. caudatus*. The membranes of *Colpodella* sp. (ATCC 50594) surround the membrane and cytoplasm of *P. caudatus*. Thickening of the pellicle is shown with the three black arrows on either side of the tether. aF, anterior flagellum; B, bacteria; m, mitochondria. Scale bars, (**A**) 2 µm, (**B**) 0.5 µm, (**C**) 500 nm and (**D**) 200 nm.

**Figure 8:**
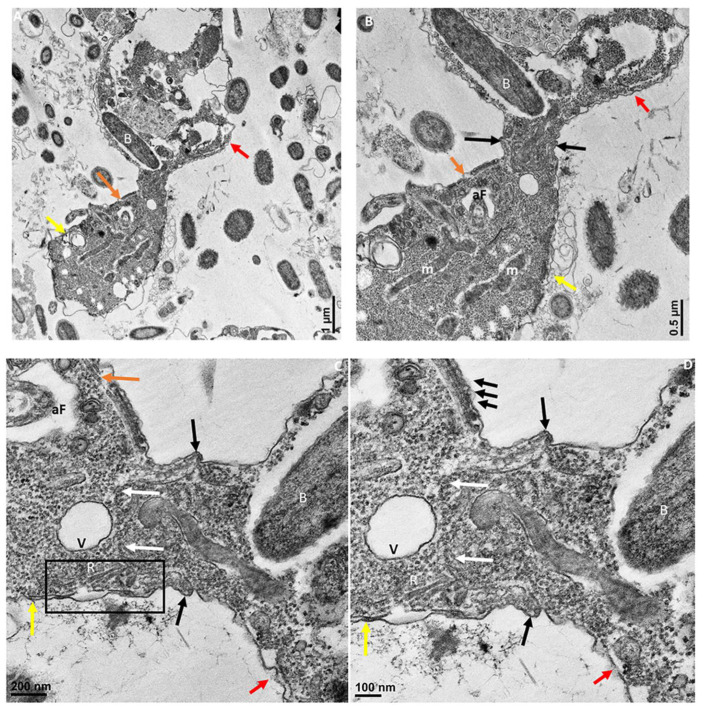
Transmission electron microscopy showing attachment of *Colpodella* sp. (ATCC 50594) (yellow arrow) to *P. caudatus* (red arrow). Panel (**A**) is enlarged to show details of attachment and tubular tether in panels (**B**–**D**). Orange arrow shows apical tip of rostrum. The attachment site is indicated by black arrows and shows the membrane of *Colpodella* sp. (ATCC 50594) trophozoite surrounding the plasma membrane and cytoplasm of aspirated *P. caudatus* (panel (**B**)). The progression of aspirated contents, organelles, and plasma membrane of *P. caudatus* is indicated by the white arrows. Thickened pellicle of *Colpodella* sp. (ATCC 50594) and microtubular organization is shown by the triple arrows. An area posterior to the apical tip of the rostrum forms an “aperture” or duct that surrounds and pulls in the plasma membrane and cytoplasmic contents of the prey (black arrows). aF, anterior flagellum; B, bacteria; m, mitochondria; R, rhoptries; V, vacuole. Scale bars, (**A**) 1 µm, (**B**) 0.5 µm, (**C**) 200 nm, and (**D**) 100 nm.

In Figure 6B, the attachment site shown in panel A is enlarged to show two points of attachment (white arrows) between the membrane of *Colpodella* sp. (ATCC 50594) trophozoites and the membrane of *P. caudatus* (boxed area). Bacteria (B) and mitochondria (m) present in the cytoplasm of *P. caudatus* were identified. We showed previously [24], and confirmed in the present study, that attachment is a two-step process. The initial attachment of *Colpodella* sp. (ATCC 50594) does not lead to penetration of the plasma membrane of *P. caudatus*. Attachment to the prey’s plasma membrane is followed by engulfment of the prey’s membrane into the cytoplasm of the predator, to facilitate dissolution of the membrane to allow the flow of cytoplasmic contents into the predator’s cytoplasm. Figure 6 shows the earliest step of the attachment process. Previous reports of myzocytosis in *Colpodella* species showed the steps after the initial contact between predator and prey [6,7,8,23]. Brugerolle [6] reported the formation of a channel-like structure at the site of attachment through which contents of the prey were aspirated into the cytoplasm of *Colpodella vorax.* A clear opening was reported between the attached trophozoites of *Colpodella gonderi* and the prey *Colpoda fastigata* [23] similar to our earlier observations [24]. In previous studies, we showed that the initial contact is followed by the microtubular organization at the point of contact between predator and prey [24]. This was also reported in *Colpodella vorax*, *C. gonderi*, and *C. tetrahymenae* [6,7,23]. Transmission electron microscopy of the tubular tether formed during myzocytosis is shown in Figure 7 and Figure 8. The membrane observed within the cytoplasm of *Colpodella* sp. (ATCC 50594) is the plasma membrane of *Parabodo caudatus* (white arrows, Figure 7B–D and Figure 8C,D). An aperture or duct opens up to engulf the prey’s membrane. Cavaliar-Smith and Chao [7] observed that there was a distance between the tip of the rostrum of *C. tetrahymenae* and the attachment site, similar to what we also show in the current and previous studies [24]. A reorientation of the attachment site to bring the tip of the rostrum in direct contact with the prey, as reported with *C. tetrahymenae* [7], was not observed in the current study.

The enlargement of the tubular tether in Figure 7B–D and Figure 8B–D shows the details of the tether at the attachment site. The tether initially consists of the membrane of *Colpodella* sp. (ATCC 50594) surrounding and engulfing the plasma membrane and cytoplasm of *P. caudatus*. Pellicular microtubular organization at the point of contact supports the tether as the prey’s cytoplasm is pulled into the cytoplasm of *Colpodella* sp. (ATCC 50594) (black arrows). The thickening of the pellicle in the predator is shown by the three arrows on either side of the tether (Figure 7C,D). An area posterior to the apical tip of the rostrum forms an aperture or duct with the membranes of the predator encircling the prey’s plasma membrane, and on either side of the attachment site, the membrane is shown as extended rims around the prey.

**Figure 9:**
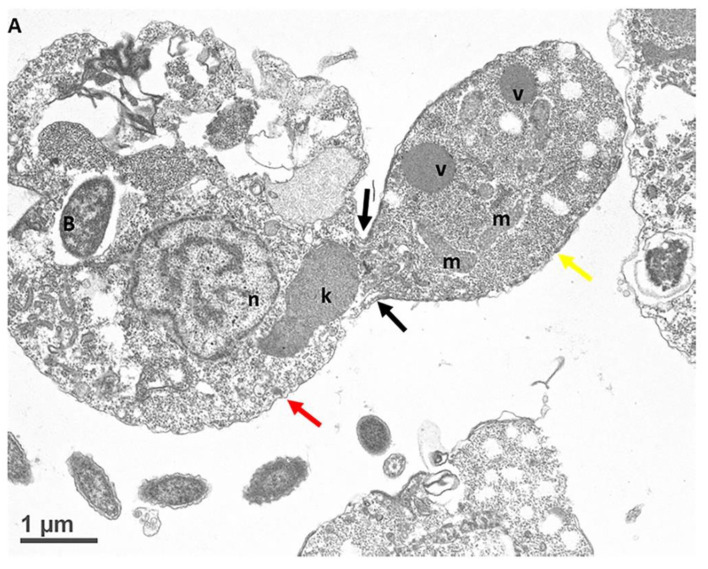

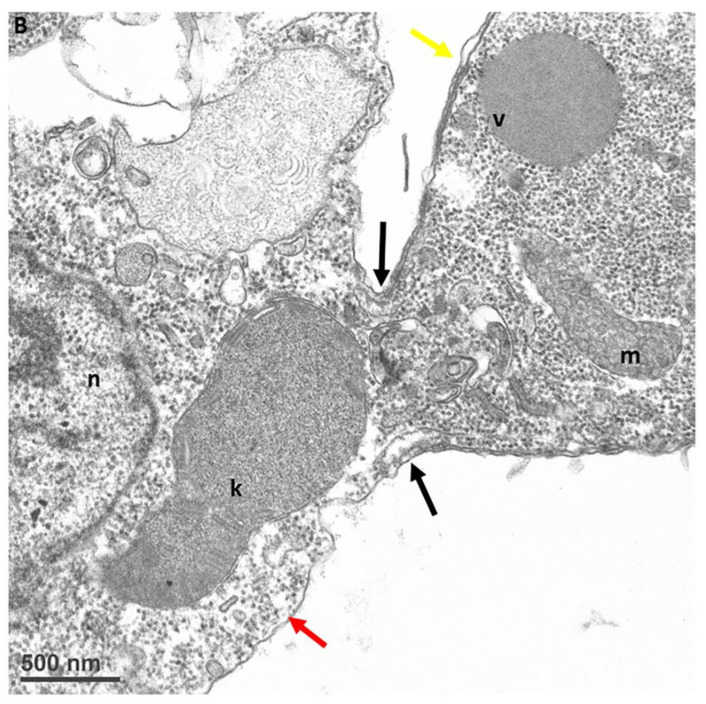
Transmission electron microscopy showing attachment of *Colpodella* sp. (ATCC 50594) (yellow arrow) to *P. caudatus* (red arrow). Panel (**A**) shows attachment of a *Colpodella* sp. (ATCC 50594) trophozoite to *P. caudatus*. Contents from the prey flow into the cytoplasm of the predator. Panel (**B**) is enlarged from panel (**A**) to show details of the attachment and tubular tether. The attachment site is indicated by black arrows and shows that the rims of the membrane of *Colpodella* sp. (ATCC 50594) trophozoite surround the opening created by dissolution of the plasma membrane of the prey. k, kinetoplast; m, mitochondria; n, nucleus; v, vesicle. Scale bars, (**A**) 1 µm, (**B**) 500 nm.

The plasma membrane of the prey is destroyed by enzymes and secreted contents from the apical complex organelles allowing for cytoplasmic contents from the prey to flow into the cytoplasm of the predator (Figure 9A and enlarged in Figure 9B). The rhoptries visible around the attachment site (Figure 8C,D) can no longer be seen in Figure 9A,B, suggesting that contents of the apical complex organelles participate in myzocytosis. Electron microscopy micrographs show that the process of myzocytosis in *Colpodella* sp. (ATCC 50594) does not occur at the apical tip of the rostrum with a piercing of the plasma membrane of *P. caudatus*. We show in the current study that the process of myzocytosis in *Colpodella* sp. (ATCC 50594) is sequential and may involve signal transduction and the release of enzymes and secreted molecules from the rhoptries and micronemes to digest the plasma membrane of the prey, including macromolecules and organelles from the prey’s cytoplasm, similar to events in the pathogenic apicomplexan [4]. The *Colpodella* sp. (ATCC 50594) trophozoite shown in Figure 9 is in the early process of aspirating cytoplasmic contents of the prey as a food vacuole is not yet visible.

**Figure 10:**
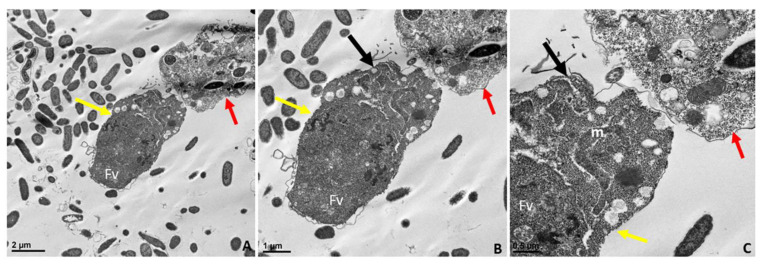
Transmission electron microscopy of the pre-cyst stage of *Colpodella* sp. (ATCC 50594) (**A**). The pre-cyst stage is shown enlarged in panels (**B**,**C**) and shows the disintegration of the apical end and loss of the anterior end of the cell after feeding. The food vacuole (Fv) becomes enlarged during myzocytosis before cyst formation. Scale bars, (**A**) 2 µm, (**B**) 1 µm, (**C**) 0.5 µm.

Precyst stages of *Colpodella* sp. (ATCC 50594) (Figure 10A, yellow arrow) enlarged (Figure 10B,C) to show the disintegration of the anterior end of the trophozoite are shown in Figure 10, unattached to the prey accompanied by loss of organelles and the flagella. The anterior end of the trophozoite is frayed, the integrity of the membrane is lost, and apical complex organelles are not identified. Mitochondria (m) can still be seen in the trophozoite.

**Figure 11:**
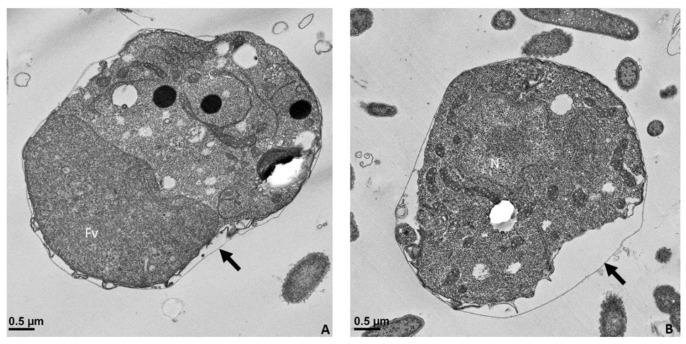

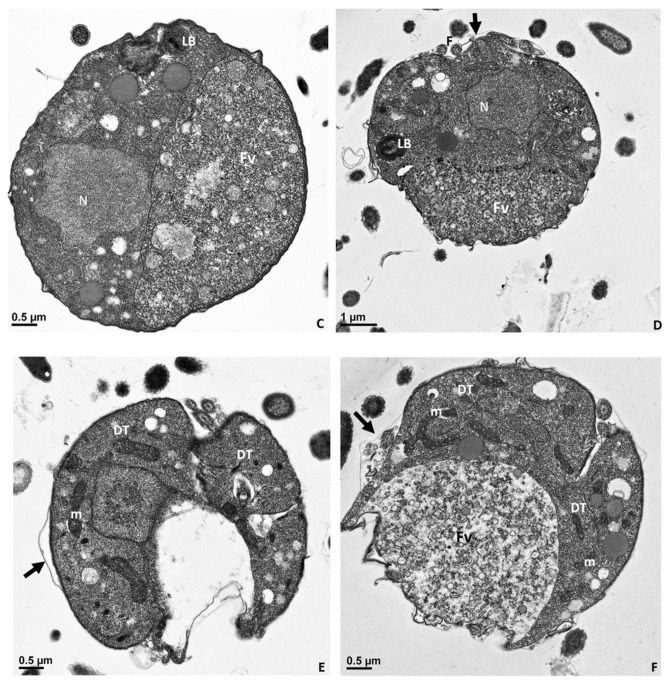

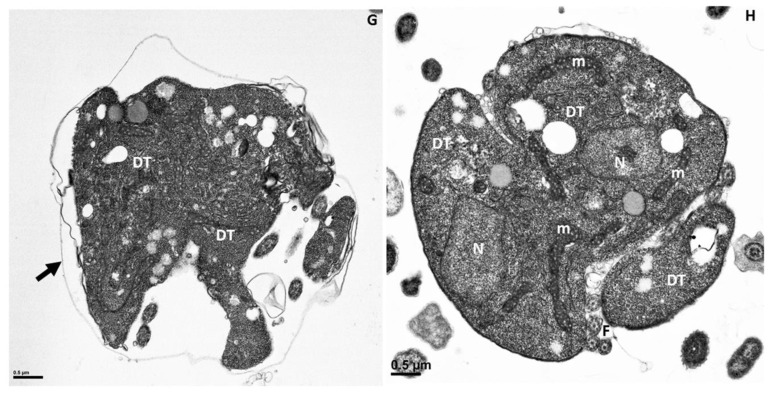
Transmission electron microscopy of cyst stages of *Colpodella* sp. (ATCC 50594). Panel (**A**) shows a young demilune cyst stage followed by single nucleus cyst stages in panels (**B**–**D**). Division of the cyst shows the developing trophozoites in panels (**E**–**G**) with a food vacuole still present as seen in panel (**F**). Three developing trophozoites (DT) are shown in panel (**H**). Flagella (F) were observed in the developing cyst. A thin cyst wall surrounds the cyst (black arrow). DT, developing cyst; F, flagella; Fv, food vacuole; LB, Lamella bodies; m, mitochondria; N, nucleus. Scale bars, (**A**–**C**), and (**E**–**H**) 0.5 µm, D, 1 µm.

The food vacuole in the cell enlarges during myzocytosis and differentiates into a cyst (Figure 11) after feeding. The young cyst representative of the demilune cyst identified by Sam-Yellowe’s trichrome stain [15] (Figure 11A) formed after the precyst is followed by a single nucleus cyst (Figure 11B–D). Remnants of the food vacuole are seen in Figure 11A,C,D,F. Mitochondria were identified in the developing trophozoites. Concentric membranes known as lamellar bodies, which may contain lipids, were identified in some cysts (Figure 11C,D, similar to observations of lamellar bodies described in *C. gonderi* trophozoites feeding on prey [23]. Cell division within the cyst occurs and shows two developing trophozoites (DT) in Figure 11E–G with the remnants of the food vacuole still present as seen in Figure 11F. Three developing trophozoites are shown in Figure 11H. Flagella (F) are observed in the developing cyst. A thin cyst wall surrounds the cyst (black arrow). The electron microscopy images show cysts of *Colpodella* sp. (ATCC 50594) with asymmetric and asynchronous division as reported previously [24].

### 2.3. Role of Actin on Colpodella sp. (ATCC 50594) and P. caudatus during Myzocytosis

**Figure 12:**
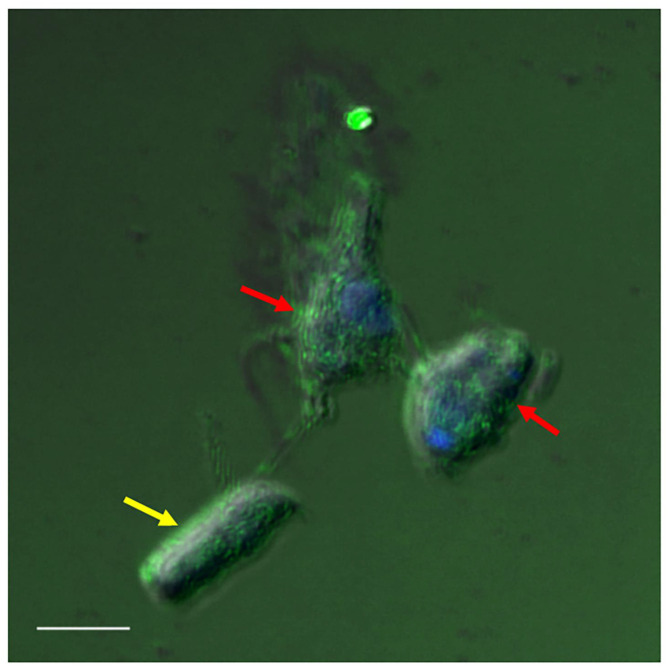
Actin-green staining of *Colpodella* sp. (ATCC 50594) (yellow arrow) and *P. caudatus* (red arrow) trophozoites. Actin is distributed in the cytoskeleton of both protists. Scale bar, 5 µm.

**Figure 13:**
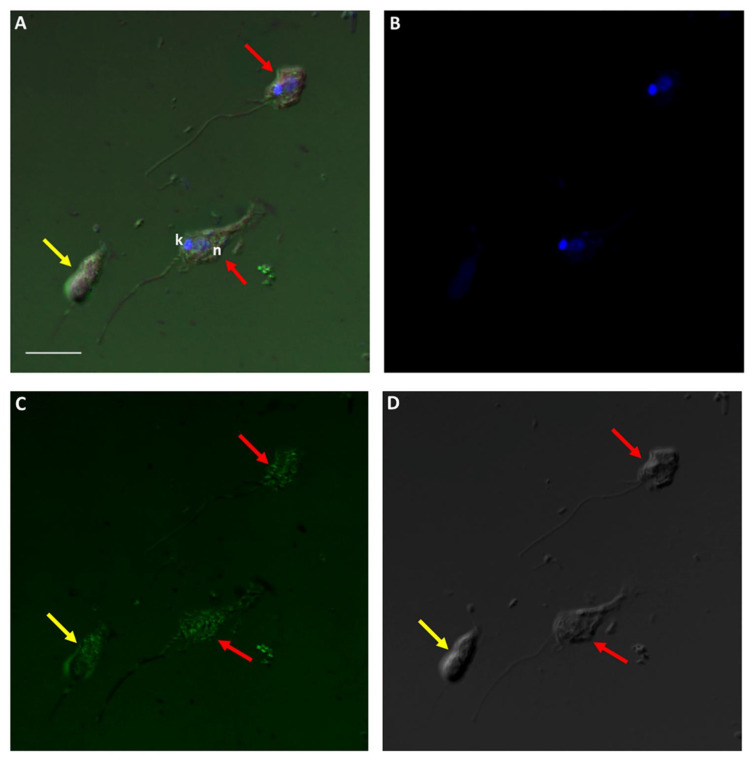
Actin-green staining of *Colpodella* sp. (ATCC 50594) (yellow arrow) and *P. caudatus* (red arrow) trophozoites for confocal microscopy and differential interference contrast (DIC) microscopy. Pre-cyst stage of *Colpodella* sp. (ATCC 50594) was identified with an enlarged food vacuole. n, nucleus; k, kinetoplast. (**A**). Overlay of actin-green, DIC, DAPI, and RhopH3 staining. (**B**). DAPI staining, (**C**). Actin-green stain, (**D**). DIC. Scale bar, 10 µm.

In order to determine the role of the cytoskeleton in the formation of the tubular tether during myzocytosis, cells from a diprotist culture were labelled with Alexa Fluor 488 phalloidin, which targets F-actin. Figure 12 and Figure 13 show the distribution of actin in the cytoskeleton of *Colpodella* sp. (ATCC 50594) and *P. caudatus* trophozoites, over the body of both cells.

**Figure 14:**
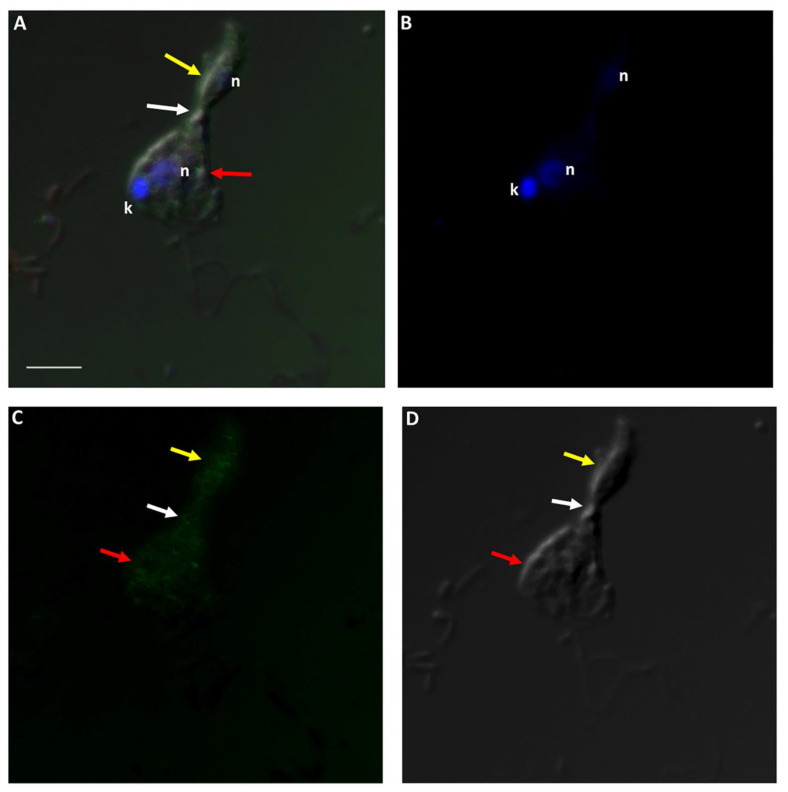
Actin-green staining of *Colpodella* sp. (ATCC 50594) (yellow arrow) and *P. caudatus* (red arrow) trophozoites in myzocytosis for confocal microscopy and differential interference contrast (DIC) microscopy. Actin green staining is distributed in the cytoskeleton of both protists and also in the area of the tubular tether (white arrow). n, nucleus; k, kinetoplast. (**A**). Overlay of actin green, DIC, DAPI, and RhopH3 staining, (**B**). DAPI staining, (**C**). Actin green staining, (**D**). DIC. Scale bar, 5 µm.

**Figure 15:**
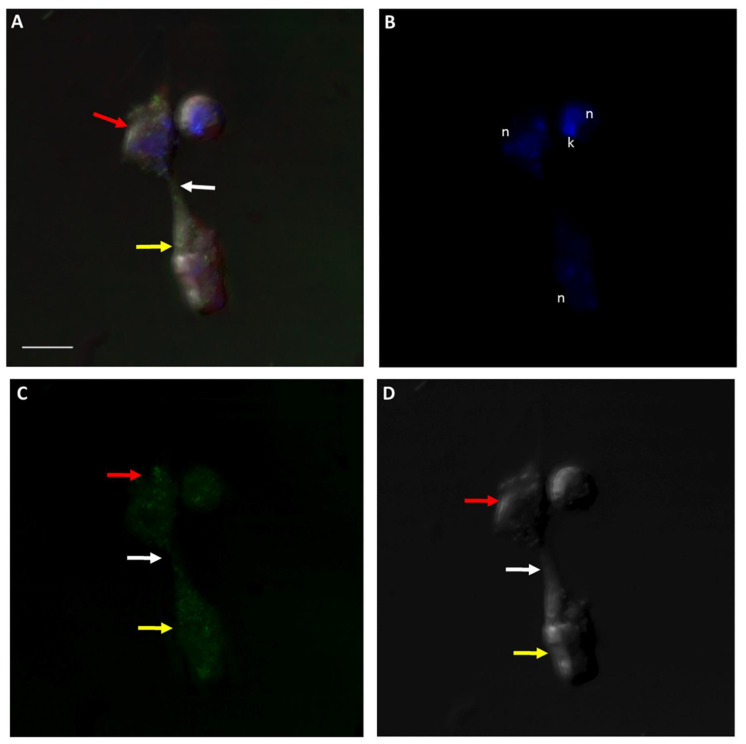
Actin-green staining of *Colpodella* sp. (ATCC 50594) (yellow arrow) and *P. caudatus* (red arrow) trophozoites in myzocytosis for confocal microscopy and differential interference contrast (DIC) microscopy. Actin green staining is distributed in the cytoskeleton of both predator and prey, in the area of the tubular tether (white arrow), and in the cyst of *P. caudatus*. n, nucleus; k, kinetoplast. (**A**). Overlay of actin green, DIC, DAPI, and RhopH3 staining. (**B**). DAPI staining, (**C**). Actin-green staining, (**D**). DIC. Scale bar, 5 µm.

A Volocity video prepared from a 3D reconstruction of Z-stacks from Figure 13 shows the actin distribution on trophozoites of predator and prey against the background of RhopH3 antibody staining (Supplementary Figure S1). Figure 14 and Figure 15 show actin staining of trophozoites in myzocytosis. Actin staining is distributed in the cytoskeleton of both protists and in the area of the tubular tether (white arrow). A Volocity video prepared from a 3D reconstruction of Z-stacks in Figure 15 (Figure S2) shows the actin distribution in the cytoskeleton of both predator and prey as shown in Figure 15C. Cells in the diprotist culture were treated with an actin polymerization inhibitor, cytochalasin D, to gain insight into the role of actin in the formation of the tubular tether.

**Figure 16:**
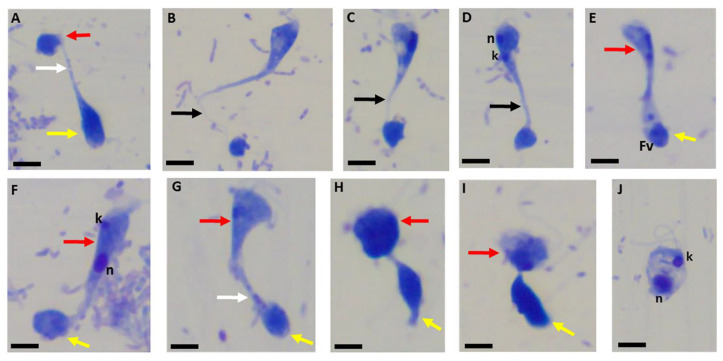
Giemsa staining of CytoD-treated cells from diprotist culture. Morphological distortions were observed in both trophozoites of *Colpodella* sp. (ATCC 50594) (yellow arrows) and *P. caudatus* (red arrows). Distorted tethers stretched between predator (yellow arrow) and prey (red arrow) are shown in panels (**A**–**G**). In some distorted tethers, round grainy material was observed in the tubular tethers (white arrows, panels (**A**,**G**)). Black arrows identify the tubular tethers. Trophozoites in myzocytosis were also observed with “normal” morphology as shown in panels (**H**,**I**). Kinetoplast (k) and nucleus (n) of *P. caudatus* appear to be stretched apart (panel (**J**)). Scale bars, 10 µm.

Volocity videos show that the distribution of actin is not limited to the point of attachment but rather present in the body of the trophozoites in both predator and prey. In order to determine the role of actin in the formation of the tether in the present study, diprotist culture was treated with cytochalasin D. Treatment of cultures with cytochalasin D resulted in morphological distortions of cells during myzocytosis (Figure 16) as well as distortions in the tubular tether (black arrows). Grainy material within the tethers was observed (white arrows) but it is not clear if they represent aspirated contents from the prey.

**Figure 17:**
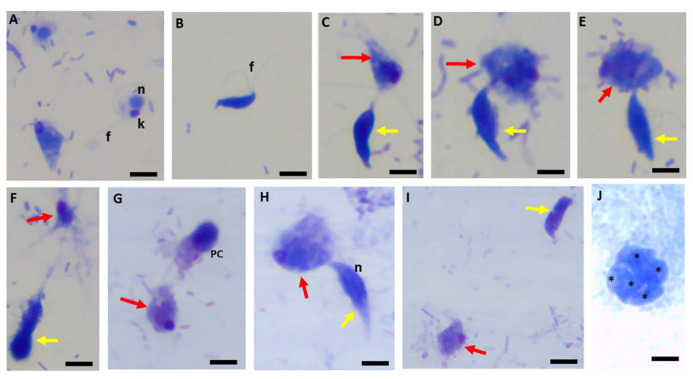
Untreated controls (panels (**A**–**F**)) show normal morphologies of *P. caudatus* trophozoites (panel (**A**)) and *Colpodella* sp. (ATCC 50594) trophozoite (panel (**B**)). Yellow arrows indicate *Colpodella* sp. (ATCC 50594), and red arrows indicate *Parabodo caudatus*. DMSO-treated controls in panel (**H**) show a trophozoite in myzocytosis. Panel (**G**) shows a precyst (PC) of *Colpodella* sp. (ATCC 50594) and trophozoite of *P. caudatus* (red arrows). Panel (**H**) shows a trophozoite of *Colpodella* sp. (ATCC 50594) (yellow arrow) in myzocytosis with *P. caudatus* (red arrow). Panel (**I**) shows trophozoites of *Colpodella* sp. (ATCC 50594) (yellow arrow) and *P. caudatus* (red arrow). Panel (**J**) shows a mature cyst of *Colpodella* sp. (ATCC 50594) with five juveniles (black asterisks). n, nucleus; k, kinetoplast; f, flagella. Scale bars, 10 µm.

Cells in myzocytosis that appeared “normal” were also observed (Figure 16H,I). The kinetoplast and nucleus of a *P. caudatus* trophozoite shown in panel J appear stretched apart. It is currently unclear why all cells were not equally affected by cytochalasin D treatment. These types of studies have not been performed with other *Colpodella* species or other free-living relatives of the Apicomplexa. This is the first study to investigate the role of actin during myzocytosis in *Colpodella* species. Our hypothesis was that motility would be affected and that attachments would not occur following treatment with cytochalasin D. However, the results obtained showed that attachments did occur with distortions to the tubular tether following cytochalasin D treatment, suggesting actin involvement in the attachment process. Differences in life cycle stages and the duration of the attachment between predator and prey may account for the differences observed. Additional investigations are needed to understand the role of the cytoskeleton in tether formation. Diprotist cultures are asynchronous at different life cycle stages and may account for the differences observed. The actinmyosin motor is essential for motility and host cell invasion among zoites of pathogenic Apicomplexa such as in *Toxoplasma gondii* and *Plasmodium* species [27]. Actin is required for zoite invasion of host cells among the Apicomplexa [2,3]. The progression of invasion through the junction formed following the attachment of merozoites to host cell erythrocytes is blocked when *Plasmodium falciparum* cultures are treated with cytochalasin B [28] and when *Toxoplasma gondii* cultures are treated with cytochalasin D [3]. Actin plays a role in the motility of *Gregarina garnhami* [29]. The treatment of *G. garnhami* with cytochalasin D resulted in the inhibition of motility [29]. The use of other drugs targeting actin will be employed to understand the role of actin in tether formation. Untreated controls (Figure 17A–F) and DMSO controls (Figure 17G–J) showed normal cell, cyst, and tether morphology (Figure 17).

*Parabodo caudatus* trophozoites (Figure 17A), *Colpodella* sp. (ATCC 50594) trophozoites (Figure 17B), and other life cycle stages including cells in myzocytosis, Figure 17H, showed normal morphology. Mature cysts of *Colpodella* sp. (ATCC 50594), Figure 17J were also observed in DMSO controls. We show that the steps of myzocytosis in *Colpodella* sp. (ATCC 50594) are sequential and resemble the steps described for trophozoite stages in Archigregarines where the mucron attaches to the host cell and aspirates the host cell cytoplasm using myzocytosis [30], as well as in zoites of pathogenic Apicomplexa. Our data show that initial contact between *Colpodella* sp. (ATCC 50594) trophozoites and *P. caudatus* is followed by the engulfment of the plasma membrane and cytoskeleton of *P. caudatus* forming the tubular tether between predator and prey. Destruction of the prey’s membrane by enzymes from the predator leads to the formation of an open channel through which the prey’s cytoplasmic contents are aspirated into the predator. Myzocytosis in *Colpodella* sp. (ATCC 50594) may serve as the immediate precursor to myzocytosis observed among the gregarines and *Cryptosporidium* species [14,30]. Additional studies will be required to understand the mechanism of the initial stages of attachment between *Colpodella* sp. (ATCC 50594) trophozoites and *P. caudatus.* Future ultrastructural studies will clarify the cytoskeletal contributions to the different lengths of the tubular tethers observed. The identification of molecules participating in myzocytosis will provide important insights to aid in the determination of the origins of the invasion and nutrition acquisition mechanisms among zoites of pathogenic apicomplexans.

## 3. Materials and Methods

### 3.1. Hay Medium Cultures

*Colpodella* sp. (ATCC 50594) was maintained in the Hay medium as a diprotist culture containing *Parabodo caudatus* in the supplied culture. The Hay medium was bacterized with *Enterobacter aerogenes* in tissue culture flasks as described [31]. Ten milliliters of culture were maintained in T25 flasks and 30 mL in T75 flasks. *Parabodo caudatus* in the diprotist culture served as prey for *Colpodella* sp. (ATCC 50594). Cultures were examined using an inverted microscope to observe different stages of trophozoites and cysts as described [32].

### 3.2. Fixation of Cells

*Colpodella* sp. (ATCC 50594) in diprotist cultures was fixed using 5% formalin as described [33]. Briefly, a viable diprotist culture was mixed with equal volumes of 10% formalin directly in the culture flask and incubated for ten minutes at room temperature [31]. The fixed cells were scraped gently with a cell scraper and the cells were transferred to a 50 mL centrifuge tube for centrifugation as described previously [31]. Following centrifugation, the supernatant was discarded, and the pellets were resuspended in 1X dPBS and centrifuged. The dPBS supernatant was discarded, and the pellets were resuspended in 100 μL of PBS. Ten microliters of cells were placed on glass slides to prepare smears. The slides were air-dried at room temperature and then used for staining protocols.

### 3.3. Actin Green488 Stain and Immunofluorescence Assay

For direct staining of F-actin with a fluorescent label, formalin-fixed *Colpodella* sp. (ATCC 50594) from a diprotist culture was permeabilized for 5 min in 10 mL of 0.1% Triton-X100 in Dulbecco’s PBS (dPBS). Slides were washed twice with dPBS and blocked in 3% bovine serum albumin for 30 min. Following 2 washes in dPBS, the ActinGreen 488 Ready Probes reagent (Invitrogen by Thermo Fisher Scientific, Grand Island, NY, USA) was applied to slides and incubated for 30 min in a covered humid chamber protected from light following manufacturer’s instructions. Slides were washed in dPBS followed by the addition of DAPI (4′, 6-diamidino-2-phenylindole) mounting solution to the smear and the application of a cover slip. The edges of the coverslip were sealed with clear nail polish. For the application of antibodies to actin488-labeled smears for immunofluorescence assay (IFA) colocalization studies, diluted primary antibodies were applied to the slides after ActinGreen 488 staining and dPBS washes. The Anti-RhopH3 [34] primary antibody was incubated with smears for 1 h at room temperature (RT) in a humid chamber, followed by 3 washes with dPBS in a coplin jar. A diluted secondary antibody conjugated with ALEXA 547 (red) was applied to the smear and incubated for 1 h in a humid chamber at RT. Slides were washed 3x in dPBS in a coplin jar and mounted with fluoroshield (Abcam) containing DAPI (4′, 6-diamidino-2-phenylindole) or Fluoromount-G (Southern Biotech, Birmingham, AL, USA) for confocal microscopy. Smears were sealed with clear nail polish for permanent storage. IFA slides were examined at the imaging core (Learner Research Institute, Cleveland Clinic). Confocal, fluorescent, and differential interference contrast (DIC) images were collected using a Leica SP8 True Scanning Confocal (TCS) DM18 inverted microscope (Leica Microsystems, GmbH, Wetzlar, Germany). Stained and confocal images were adjusted to 300 dpi using the CYMK color mode, RGB color mode, auto color, and auto contrast on Adobe Photoshop (CC). 3D reconstructions of confocal z-stacks were performed using Volocity v.6.3.0 software (Quorum Technologies Inc., Puslinch, ON, Canada).

### 3.4. Treatment of Colpodella sp. (ATCC 50594) in Diprotist Culture with Cytochalasin D

Cells in diprotist Hay medium culture were treated with cytochalasin D (cytoD) (Sigma Aldrich). Cells cultured in 24- or 6-well microtiter plates or T25 tissue flasks were treated with the drug reconstituted in dimethyl sulfoxide (DMSO). Cells were incubated with Cyto D at 1 µM, 2.5 µM, 5 µM, and 10 µM in duplicate in microtiter plates. Three independent experiments were performed and then repeated at concentrations of 10 µM. CytoD was added to cells subcultured from resting cyst stages, scraped with cell scrapers, and inoculated into a bacterized Hay medium (treatment before excystation), or cytoD was added into actively growing cultures with young trophozoites undergoing myzocytosis (treatment after excystation). Cultures incubated with DMSO at the same volumes and untreated cells served as controls. Cultures were monitored by observing cells under an inverted microscope. At 48 h, cells were fixed in 5% formalin, collected, and centrifuged. Supernatants were discarded, the pellets were washed in 1x dPBS, and smears were prepared on glass slides for the Giemsa stain. DMSO-treated cells and untreated control cells were observed in culture during the 48 h incubation period, then formalin-fixed and collected for centrifugation, smear preparation, and staining with the Giemsa stain.

### 3.5. Light Microscopy

For light microscopy, formalin-fixed cells were stained with Giemsa, Kinyoun’s carbol fucshin, and Sam-Yellowe’s trichrome stains [32]. All stained smears were examined under oil immersion at x1000 magnification and images were captured using an Olympus BX43 compound microscope attached to an Infinity HD Lumenera digital camera and Olympus U-TV0.35xc-2 adapter using Infinity HD Capture software.

### 3.6. Transmission Electron Microscopy

*Colpodella* sp. (ATCC 50594) in the diprotist culture was harvested and fixed as described previously [24]. After embedding in an EMbed 812 embedding media (Electron Microscopy Sciences, Hatfield, PA, USA), thin sections (70 nm) were cut on an RMC MT6000-XL ultramicrotome, mounted on T-300 mesh nickel grids (Electron Microscopy Sciences, Hatfield, PA, USA), and then sequentially stained with acidified methanolic uranyl acetate and stable lead staining solution. These were coated on a Denton DV-401 carbon coater (Denton Vacuum LLC, Moorestown, NJ, USA), and observed in an FEI Tecnai Spirit (T12) transmission electron microscope with a Gatan US4000 4kx4k CCD.

## Data Availability

Not applicable.

## Supplementary Materials

The following supporting information can be downloaded at: https://www.mdpi.com/article/10.3390/pathogens11040455/s1, Figure S1: Volocity video showing actin distribution on trophozoites of Colpodella sp. (ATCC 50594) and P. caudatus; Figure S2: Volocity video showing actin distribution on trophozoites of Colpodella sp. (ATCC 50594) and P. caudatus in myzocytosis.

## References

[1] Bargieri D., Lagal V., Andenmatten N., Tardieux I., Meissner M., Ménard R. (2014). Host cell invasion by apicomplexan parasites: The junction conundrum. PLoS Pathog..

[2] Del Rosario M., Periz J., Pavlou G., Lyth O., Lattore-Baaragan F., Das S., Pall G.S., Stortz J.F., Lemgruber L., Whitelaw J.A. (2019). Apicomplexan F-actin is required for efficient nuclear entry during host cell invasion. EMBO Rep..

[3] Drewry L.L., Sibley L.D. (2015). *Toxoplasma* actin is required for efficient host cell invasion. MBio.

[4] Gubbels M., Duraisingh M.T. (2012). Evolution of apicomplexan secretory organelles. Int. J. Parasitol..

[5] Simpson A., Patterson D. (1996). Ultrastructure and identification of the predatory flagellate *Colpodella pugnax* Cienkowski (Apicomplexa) with a description of *Colpodella turpis* n. sp. and a review of the genus. Syst. Parasitol..

[6] Brugerolle G. (2002). *Colpodella vorax*: Ultrastructure, predation, life-cycle, mitosis, and phylogenetic relationships. Eur. J. Protistol..

[7] Cavalier-Smith T., Chao E. (2004). Protalveolate phylogeny and systematics and the origins of Sporozoa and dinoflagellates (phylum Myzozoa nom. nov.). Eur. J. Protistol..

[8] Mylnikova Z.M., Mylnikov A.P. (2009). The morphology of predatory flagellate *Colpodella pseudoedax*. Inland Water Biol..

[9] Fussy Z., Masarova P., Krucinska J., Esson H.J., Obornik M. (2017). Budding of the Alveolate alga *Viterella brassicaformis* resembles sexual and asexual processes in Apicomplexan parasites. Protist.

[10] Okamoto N., Keeling P.J. (2014). The 3D structure of the apical complex and association with the flagellar apparatus revealed by serial TEM tomography in *Psammosa pacifica*, a distant relative of Apicomplexa. PLoS ONE.

[11] Okamoto N., Horak A., Keeliong P.J. (2012). Description of two species of early branching Dinoflagellates, *Psammosa pacifica* n. g., sp. and *P. atlantica* n. sp.. PLoS ONE.

[12] Obornik M., Vancova M., Lai D., Janouskovec J., Keeling P.J., Lukes J. (2011). Morphology and ultrastructure of multiple life cycle stages of the photosynthetic relative of apicomplexan, *Chromera velia*. Protist.

[13] Schnepf E., Deichgraber G. (1984). “Myzocytosis”, a kind of endocytosis with implications to compartmentation in endosymbiosis. Naturwissenschaften.

[14] Valigurova A., Florent I. (2021). Nutrient acquisition and attachment strategies in basal lineages: A tough nut to crack in the evolutionary puzzle of apicomplexan. Microorgaisms.

[15] Sam-Yellowe T.Y., Getty T.A., Addepalli K., Walsh A.M., Williams-Medina A.R., Fujioka H., Peterson J.W. Novel Life Cycle Stages of *Colpodella* sp. (Apicomplexa) Identified Using Sam-Yellowe’s Trichrome Stains and Confocal and Electron Microscopy. https://link.springer.com/article/10.1007/s10123-021-00175-z.

[16] Yuan C.L., Keeling P.J., Krause P.J., Horak A., Bent S., Rollend L., Hua X.G. (2012). *Colpodella* spp.-like parasite infection in woman, China. Emerg. Infect. Dis..

[17] Jiang J.F., Jiang R.R., Chang Q.C., Zheng Y.C., Jiang B.G., Sun Y., Jia N., Wei R., Liu H.B., Huo Q.B. (2018). Potential novel tick-borne *Colpodella* species parasite infection in patient with neurological symptoms. PLoS Negl. Trop. Dis..

[18] Neculicioiu V.S., Colosi I.A., Toc D.A., Lesan A., Costache C. (2021). When a ciliate meets a flagellate: A rare case of *Colpoda* spp. and *Colpodella* spp. isolated from the urine of a human patient. Case report and brief review of the literature. Biology.

[19] Matsimbe A.M., Magaia V., Sanchez G.S., Neves L., Noormahomed E., Antunes S., Domingos A. (2017). Molecular detection of pathogens in ticks infesting cattle inNampula province, Mozambique. Exp. Appl. Acarol..

[20] Squarre D., Nakamura Y., Hayashida K., Kawai N., Chambaro H., Namangala B., Sugimoto C., Yamagishi J. (2020). Investigation of the piroplasm diversity circulating in wildlife and cattle of the greater Kafue ecosystem, Zambia. Parasite Vectors.

[21] Solarz W., Najberek K., Wilk-Wozniak E., Biedrzycka A. (2020). Raccoons foster the spread of freshwater and terrestrial microorganisms-mammals as source of microbial eDNA. Divers. Distrib..

[22] Hussein S., Li X., Bukharr S.M., Zhou M., Ahmad S., Amhad S., Javid A., Guan C., Hussain A., Ali W. (2021). Cross-genera amplification and identification of *Colpodella* sp. with *Cryptosporidium* primers in fecal samples of zoo felids from northeast China. Braz. J. Biol..

[23] Foissner W., Foissner I. (1984). First record of an ectoparasitic flagellate on ciliates: An ultrastructural investigation of the morphology and the mode of attachment of *Spiromonas gonderi* Nov. Spec. (Zoomastigophora, Spiromonadidae) invading the pellicle of ciliates of the genus *Colpoda* (ciliophoran, Colpodidae). Protistologica.

[24] Getty T., Peterson J.W., Fujioka H., Walsh A.M., Sam-Yellowe T.Y. (2021). *Colpodella* sp. (ATCC 50594) Life Cycle: Myzocytosis and Possible Links of the origin of intracellular parasitism. Trop. Med. Infect. Dis..

[25] Mylnikov A.P. (2009). Ultrastructure and phylogeny of colpodellids (*Colpodellida*, Alveolata). Biol. Bull..

[26] Myl’nikov A.P., Myl’nikova Z.M. (2008). Feeding spectra and pseudoconoid structure in predatory alveolate flagellates. Inland Water Biol..

[27] Soldati-Favre D. (2008). Molecular dissection of host cell invasion by the apicomplexans: The glideosome. Parasite.

[28] Miller L.H., Aikawa A., Johnson J.G., Shiroishi T. (1979). Interaction between cytochalasin B-treated malarial parasites and erythrocytes. Attachment and junction formation. J. Exp. Med..

[29] Kovacikova M., Vaskovicova N., Nebesarova J., Valigurova A. (2018). Efffect of jasplakinolide and cytochalasin D on cortical elements involved in the gliding motility of the eugregarine *Gregarina garnhami* (Apicomplexa). Eur. J. Protistol..

[30] Paskerova G.G., Miroliubova T.S., Diakin A., Kovacikova M., Valigurova A., Guillou L., Aleoshin V.V., Simdyanov T.G. (2018). Fine structure and molecular phylogenetic position of two marine gregarines, *Selenidium pygospionis* sp. n. and *S. pherusae* sp. n., with notes on the phylogeny of Archigregarinida (Apicomplexa). Protist.

[31] Sam-Yellowe T., Yadavalli R. (2018). Giemsa Staining and Antibody Characterization of *Colpodella* sp. (Apicomplexa). Microbiol. Modern Tech..

[32] Yadavalli R., Sam-Yellowe T. (2017). Developmental stages identified in the trophozoite of the free-living Alveolate flagellate *Colpodella* sp. (Apicomplexa). Int. Microbiol..

[33] Sam-Yellowe T., Addepalli K., Yadavalli R., Peterson J.W. (2019). New trichrome stains identify cysts of *Colpodella* sp. (Apicomplexa) and *Bodo caudatus*. Int. Microbiol..

[34] Yang J.C., Blanton R.E., King C.L., Fujioka H., Aikawa M., Sam-Yellowe T.Y. (1996). Seroprevalence and specificity of human responses to the *Plasmodium falciparum* rhoptry protein Rhop-3 determined by using a C-terminal recombinant protein. Infect. Immun..

